# Supplementary material for: Established rodent community delays recovery of dominant competitor following experimental disturbance
Source: Proc Biol Sci. 2019 Dec 11;286(1917):20192269. doi: 10.1098/rspb.2019.2269 (PMC6939914; doi:10.1098/rspb.2019.2269)
Supplement: Appendix: supplementary figures and tables [file rspb20192269supp1.docx]

Electronic Supplementary Material

Paper title: Established rodent community delays recovery of dominant competitor following experimental disturbance

Authors: E.M. Christensen, G.L. Simpson, S.K.M. Ernest

Journal: Proceedings of the Royal Society B

DOI: 10.1098/rspb.2019.2269

Population-level analyses

In order to better understand the observed differences in population size of kangaroo rats between treatments, we performed population-level analyses on these species during the period after the treatment change. We examined the rate that new kangaroo rat individuals entered each plot type, and performed a multistrata population model using the package RMark (version 2.2.6; [1]) to estimate survivorship (S) of kangaroo rat species on each experimental treatment type and probability of transition (Psi) between treatment types.

***New individuals***

New individuals (i.e. first-time captures) on a plot represent the combination of births that occurred on the plot and immigrants from somewhere external to the study site. While our study design does not enable us to distinguish between these two rates, the total number of first-time captures on a plot during a specified time period is nevertheless a reflection of the suitability of that plot to support reproduction and/or encourage colonization by outside individuals. For both *Dipodomys merriami* and *D. ordii,* fewer individuals were captured on kangaroo rat+ plots compared to controls, and more individuals were captured on rodent+ plots compared to controls (Table S1-S2).

**Table S1: average number of new *Dipodomys merriami* individuals per plot following treatment change**

| Treatment | Year 1 | Year 2 | Year 3 | **Total** |
| --- | --- | --- | --- | --- |
| Control | 8.75 | 9.75 | 8.0 | **26.5** |
| Kangaroo rat+ | 7.0 | 9.33 | 6.33 | **22.67** |
| Rodent+ | 12.67 | 15.0 | 5.67 | **33.33** |

**Table S2: average number of new *Dipodomys ordii* individuals per plot following treatment change**

| Treatment | Year 1 | Year 2 | Year 3 | **Total** |
| --- | --- | --- | --- | --- |
| Control | 5.25 | 2.0 | 1.0 | **8.25** |
| Kangaroo rat+ | 2.33 | 2.0 | 0.67 | **5.0** |
| Rodent+ | 5.67 | 5.0 | 3.67 | **14.33** |

***Survivorship and probability of transition between treatments***

We performed a multistrata model to estimate survivorship (S) of kangaroo rat species on each experimental treatment type and probability of transition (Psi) between treatment types. For *D. merriami,* we found that the best model included separate estimates of survivorship based on treatment type, and separate estimates of transition probabilities based on treatment type (Table S3). For *D. ordii,* we found that the best model included separate estimates of survivorship based on treatment type, but no difference in transition probabilities based on treatment type (Table S4).

**Table S3: estimates from multistrata model for *Dipodomys merriami* (based on mark-recapture data from 315 individuals)**

|  | estimate | Standard error | LCL | UCL |
| --- | --- | --- | --- | --- |
| S: long-term control | 0.859072 | 0.012446 | 0.832871 | 0.881748 |
| S: former kangaroo rat removal | 0.902081 | 0.012923 | 0.873671 | 0.924653 |
| S: former rodent removal | 0.860455 | 0.013472 | 0.831901 | 0.884830 |
| Psi: long-term control to former kangaroo rat removal | 0.003053 | 0.00216 | 0.000761 | 0.01216 |
| Psi: long-term control to former rodent removal | 0.012813 | 0.004508 | 0.006413 | 0.025435 |
| Psi: former kangaroo rat removal to long-term control | 0.008705 | 0.004341 | 0.003265 | 0.022999 |
| Psi: former kangaroo rat removal to former rodent removal | 0.006011 | 0.003621 | 0.00184 | 0.019452 |
| Psi: former rodent removal to long-term control | 0.014983 | 0.005262 | 0.0078507 | 0.029685 |
| Psi: former rodent removal to former kangaroo rat removal | 0.001804 | 0.001802 | 0.000254 | 0.012691 |

**Table S4: estimates from multistrata model for *Dipodomys ordii* (based on mark-recapture data from 96 individuals)**

|  | estimate | Standard error | LCL | UCL |
| --- | --- | --- | --- | --- |
| S: long-term control | 0.859465 | 0.021916 | 0.8108 | 0.897199 |
| S: former kangaroo rat removal | 0.773893 | 0.054854 | 0.649308 | 0.863522 |
| S: former rodent removal | 0.792193 | 0.029331 | 0.728887 | 0.843882 |
| Psi: no differences between strata | 0.006584 | 0.00294 | 0.002739 | 0.015741 |

Comparison of rodent communities by treatment: pre-2015

To better interpret the effect of the treatment change on non-kangaroo rat populations, we compared number of non-kangaroo rat individuals between treatments for the 2-year time period leading up to the treatment change. We used a Generalized Linear Mixed Model (GLMM) to analyze the effect of treatment while accounting for between-plot differences. Models were fit with a Poisson distribution. Analyses were conducted using mgcv in R (version 3.5.1; [2]) with post hoc tests using the glht() function in the multcomp package (version 1.4-8; [3]). There were significant pair-wise differences between all pairs of treatments for number of non-kangaroo rat individuals (Figure S1, Table S5). Thus we expect the experimental treatment changes in 2015 to have measurable effects on this metric.


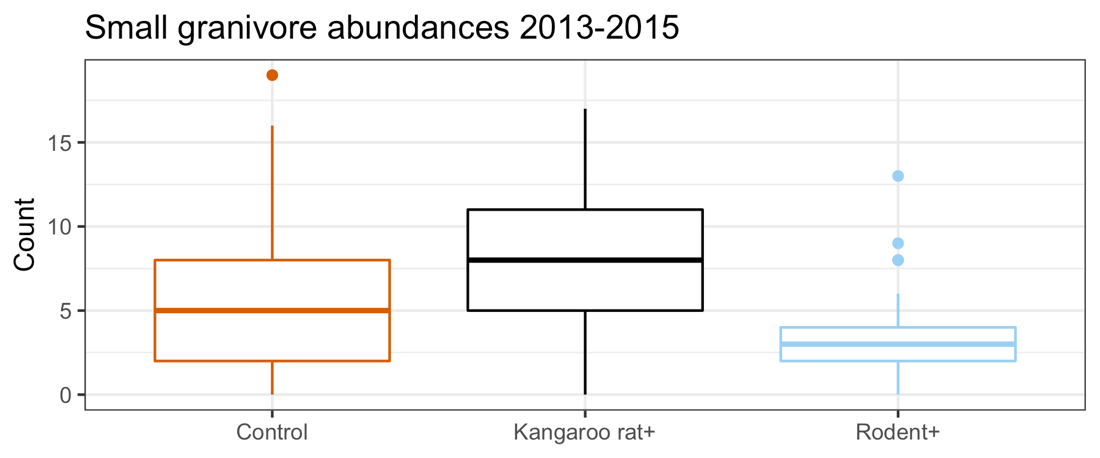


Fig. S1. Number of individuals of non-kangaroo rat species captured on plots by experimental treatment for the period before the treatment change, 2013-2015.

Table S5. Multiple comparisons of means: Tukey contrasts for number of non-kangaroo rat individuals 2013-2015.

| Treatment pair | Estimate | Std. error | Z | P |
| --- | --- | --- | --- | --- |
| Kangaroo rat removal – control | 0.3210 | 0.1346 | 2.384 | 0.0449 |
| Rodent removal – control | -0.5888 | 0.1455 | -4.047 | < 0.001 |
| Rodent removal – kangaroo rat removal | -0.9098 | 0.1519 | -5.989 | < 0.001 |

Displacement of resident individuals by invading kangaroo rats

In our analysis of the dynamics of non-kangaroo rat populations, we found that these populations quickly converged to control levels following the treatment change in 2015. If we focus on Bailey’s pocket mouse (*Chaetodipus baileyi*), the species most likely to compete directly with kangaroo rats [4,5], we see that the abundance of this species dropped quickly on former kangaroo rat removals once kangaroo rats were reintroduced (Figure S2). In fact, Bailey’s pocket mouse became extremely rare on all plot types within 9 months of the treatment change.


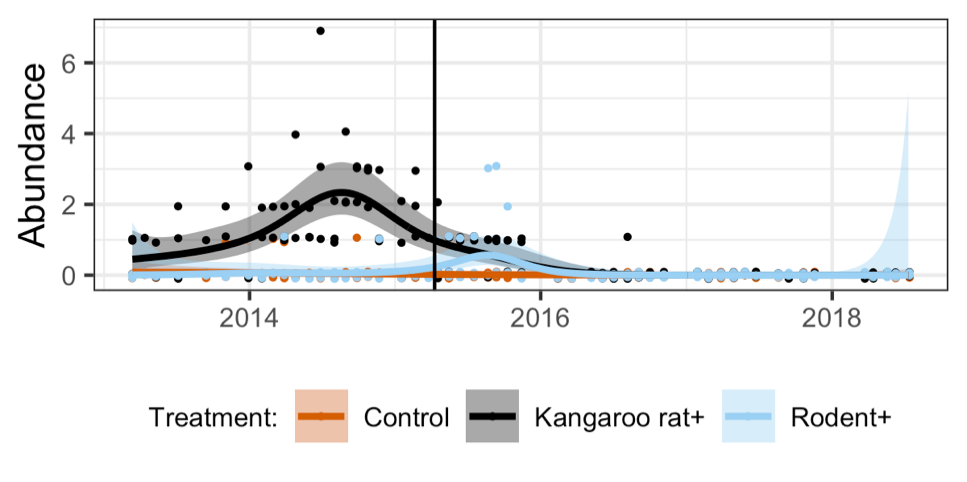


Fig. S2. Populations of Bailey’s pocket mouse on experimental plots. Solid lines are average by treatment and month. Vertical line marks when treatments were changed in March 2015.

Additional plant community comparisons

If the slight differences in plant composition made kangaroo rat removals more different from controls than the rodent removals were, then perhaps these differences could contribute to the delayed response on former kangaroo rat removals as kangaroo rats dealt with an environment that was slightly more alien than the one they were accustomed too. However, there is no evidence that this was the case. In fact the evidence suggests the opposite—when comparing rodent removals to controls, the treatment effect explains a slightly higher fraction of the variation in plant species composition (pCCA permutation test: winter annuals, R^2^_CCA_ = 0.06 and p = 0.002; summer annuals, R^2^_CCA_ = 0.05 and p = 0.002) than it does for the comparison between kangaroo rat removals and controls (pCCA permutation test: winter annuals, R^2^_CCA_ = 0.02 and p = 0.002; summer annuals, R^2^_CCA_ = 0.03 and p = 0.004; Figure S3-S4). This suggests that the plant community on the rodent removals was slightly more different from the control plots. While this does not rule out the possibility that the plant community negatively impacted the ability of kangaroo rats to reinvade former kangaroo rat removal plots, the support for this as a mechanism is weak.


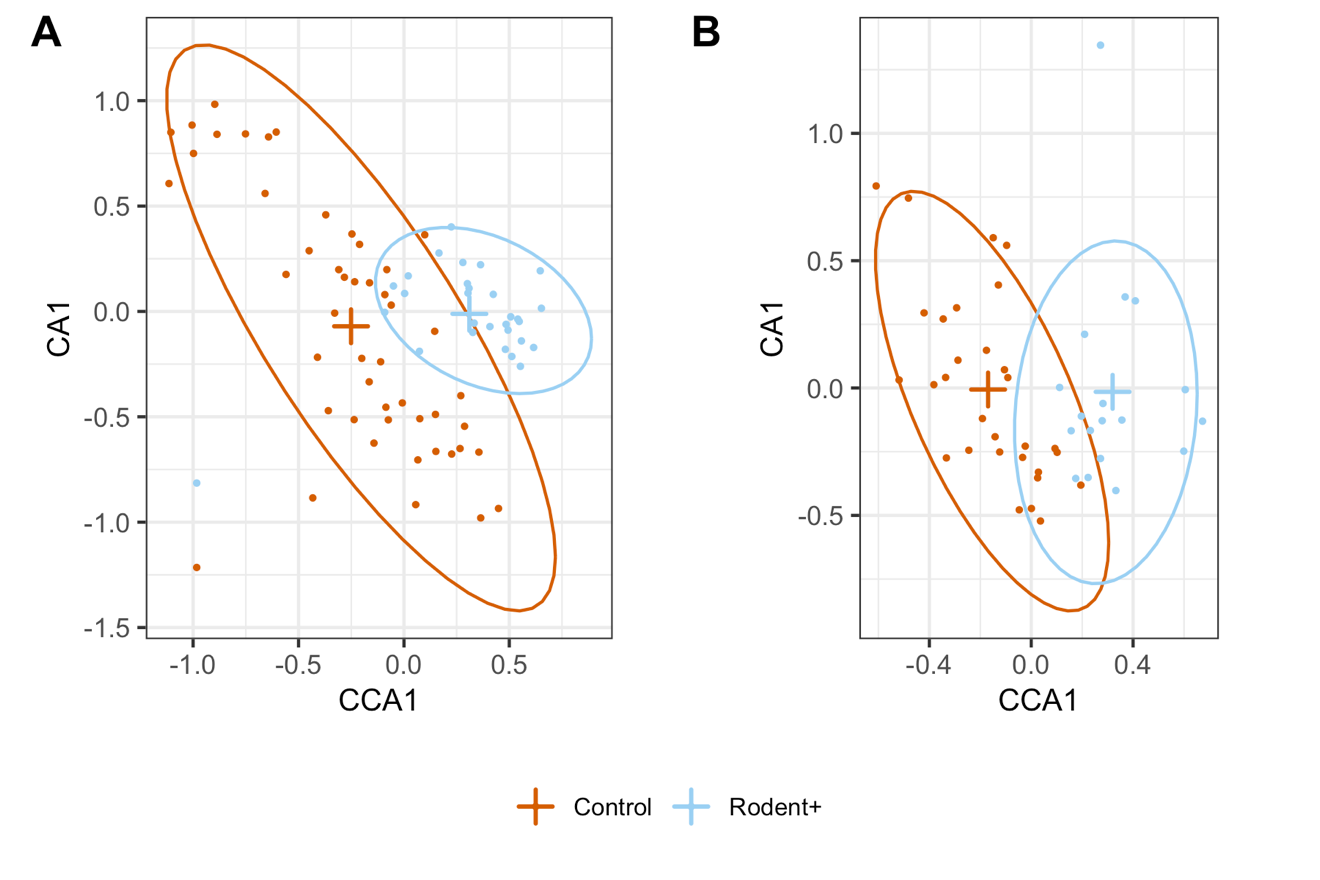


Fig. S3. Comparison of plant communities (summer and winter) on controls to plant communities on rodent removals before the treatment change in 2015.


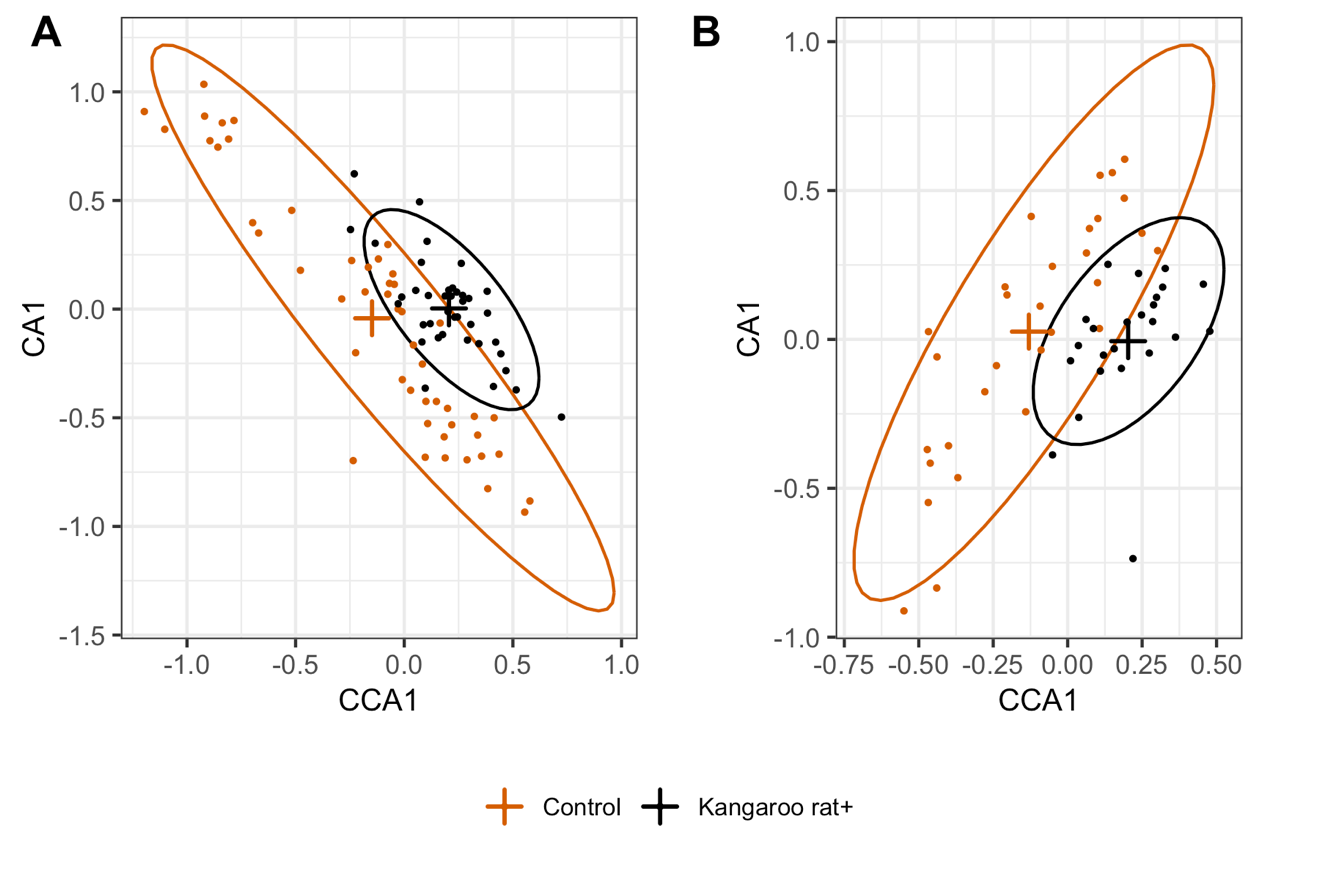


Fig. S4. Comparison of plant communities (summer and winter) on controls to plant communities on kangaroo rat removals before the treatment change in 2015.

**References**

1. Laake J. 2013 RMark: An R Interface for Analysis of Capture-Recapture Data with MARK. , 25.

2. R Core Team. 2018 *R: A language and environment for statistical computing*. Vienna, Austria: R Foundation for Statistical Computing. See https://www.R-project.org/.

3. Hothorn T, Bretz F, Westfall P. 2008 Simultaneous Inference in General Parametric Models. *Biom. J.* **50**, 346–363. (doi:10.1002/bimj.200810425)

4. Ernest SKM, Brown JH. 2001 Delayed Compensation for Missing Keystone Species by Colonization. *Science* **292**, 101–104. (doi:10.1126/science.292.5514.101)

5. Thibault KM, Ernest SKM, Brown JH. 2010 Redundant or complementary? Impact of a colonizing species on community structure and function. *Oikos* **119**, 1719–1726. (doi:10.1111/j.1600-0706.2010.18378.x)
